# The association between poor glycemic control and apical periodontitis: A systematic review and meta-analysis

**DOI:** 10.4317/medoral.27997

**Published:** 2026-01-24

**Authors:** Xueman Wang, Shanshan Guo, Xin Yu, Xulan Li, Jing Lai

**Affiliations:** 1 Department of Oral Medicine, Chongqing Dental Hospital, China

## Abstract

**Background:**

Diabetes mellitus (DM) is a common metabolic disorder, and persistent hyperglycemia may impair oral health through various immunological and inflammatory mechanisms, particularly by influencing the onset and healing of apical periodontitis (AP). This systematic review and meta-analysis aimed to evaluate whether poor glycemic control (PGC) is associated with the presence and progression of AP.

**Material and Methods:**

This study was conducted in accordance with the PRISMA 2020 guidelines. A comprehensive search of PubMed, Embase, Google Scholar, and Scopus was performed to identify relevant English-language studies published up to June 2025. Cross-sectional and longitudinal studies examining the association between glycemic control status-defined by glycated hemoglobin (HbA1c) levels-and AP-related outcomes were included. Data were synthesized using RevMan 5.3 software to calculate pooled odds ratios (ORs) and 95% confidence intervals (CIs). Heterogeneity and publication bias were also assessed.

**Results:**

A total of 18 studies comprising approximately 51,070 patients were included. The relationship between PGC and AP was examined across three domains: AP prevalence, the persistence of apical periodontitis in root-filled teeth (AP-RFT), and alterations in local or systemic immune responses. Meta-analysis of AP prevalence (6 studies) revealed that individuals with PGC had a significantly increased risk of developing AP compared to controls (OR=2.38, 95% CI: 1.98-2.86, P<0.00001). Furthermore, meta-analysis of AP-RFT persistence (8 studies) showed that patients with PGC had a significantly higher risk of AP-RFT (OR=2.74, 95% CI: 2.03-3.70, P<0.0001). Several studies also reported elevated levels of inflammatory cytokines and bacterial load in patients with PGC. Discussion: PGC appears to negatively influence both the development and healing of AP, possibly through immune-inflammatory pathways.

**Conclusions:**

PGC is closely associated with the occurrence of AP and the failure of periapical healing following endodontic treatment. PGC may aggravate periapical tissue damage and inflammation through proinflammatory immune pathways. Clinicians should consider comprehensive evaluation and individualized management of DM patients during endodontic therapy to improve treatment outcomes and oral health.

## Introduction

Diabetes mellitus (DM) is a chronic metabolic disorder characterized by persistent hyperglycemia resulting from defects in insulin secretion, insulin action, or both. According to the International Diabetes Federation (IDF), the estimated number of individuals with DM is approximately 11 million in Japan, 32.2 million in the United States, 61.4 million in Europe, and 140.9 million in China (1). The prevalence of DM in China, India, and the global average is close to 10%, while those in Japan and the United States exceed this threshold (2). Researchers project that the global number of adults with DM will increase from 463 million (9.3%) in 2019 to 578 million (10.2%) by 2030, representing a 25% rise over a decade, and further reach 700 million by 2045, reflecting a 51% increase (3). Chronic hyperglycemia in DM, resulting from insufficient insulin secretion or insulin resistance, impairs glucose uptake by peripheral tissues and leads to poor glycemic control (PGC). This persistent metabolic disturbance, coupled with systemic inflammation, contributes to the development of both microvascular and macrovascular complications, including retinopathy, cardiovascular disease, cerebrovascular disease, nephropathy, lower limb amputation (4 - 8), as well as periodontitis (9).

Glycemic control in patients with DM can be assessed using glycated protein markers, such as fructosamine, glycated albumin (GA), and glycated hemoglobin (HbA1c) (10). Fructosamine and GA reflect average blood glucose levels over the preceding 2-3 weeks and are more sensitive to short-term glycemic fluctuations, though their stability is relatively limited. In contrast, HbA1c is less affected by short-term glucose variability and protein metabolism disorders, providing a more stable and standardized indicator of average glycemic control over the past 2 to 3 months. Consequently, HbA1c is widely considered the "gold standard" for assessing long-term glycemic control in diabetic patients. Multiple large-scale prospective studies have confirmed that elevated HbA1c levels are significantly associated with an increased risk of diabetes-related complications, including retinopathy, nephropathy, neuropathy, and cardiovascular events (11 - 14). Furthermore, each 1% reduction in HbA1c has been shown to reduce the risk of microvascular complications by approximately 37%. Although the central role of HbA1c in monitoring glycemic control is well recognized, recommended target levels vary across different clinical guidelines. The American College of Physicians (ACP) suggests a more lenient target, with HbA1c levels between 7.0% and 8.0% for adult patients with DM (15). In contrast, most major organizations-including the International Diabetes Federation (IDF), the American Diabetes Association (ADA), the Chinese Diabetes Society (CDS), Diabetes Canada (DC), and the Research Society for the Study of Diabetes in India (RSSDI)-recommend a target of <7.0% (16 - 20). A joint consensus report by the ADA and the European Association for the Study of Diabetes (EASD) supports a similar target (21). More stringent targets have been proposed by the American Association of Clinical Endocrinology (AACE) and the American College of Endocrinology (ACE), which recommend HbA1c 6.5% for most patients (22). However, in certain clinical scenarios-such as in patients with renal impairment, hepatic dysfunction, or during pregnancy-HbA1c may not reliably reflect glycemic status and should be interpreted in conjunction with GA or other glycemic markers.

Apical periodontitis (AP) is an inflammatory disease characterized by apical bone resorption resulting from root canal infection. As the understanding of its etiology has advanced, recent studies have begun exploring the relationship between AP and systemic chronic diseases, though conclusive evidence remains lacking. The link between DM and periodontitis has been well established in the field of oral health, with both conditions being interrelated and mutually exacerbating (9). A common feature of both periodontitis and AP is the loss of alveolar bone. In this context, DM, particularly type 2 diabetes mellitus (T2DM), can lead to bone tissue loss or damage through disruptions in bone metabolism, a fact that is widely recognized in both clinical and basic research (23 - 24). Consequently, numerous studies in recent years have sought to confirm the association between AP and DM through clinical and animal research, although the results remain inconclusive. It is well established that when blood glucose levels are well-managed in diabetic patients, the onset of complications can be significantly delayed or even partially reversed. In contrast, PGC can exacerbate both systemic and local inflammatory responses, with the underlying biological mechanisms significantly influenced by the negative impact of hyperglycemia.

Existing studies primarily focus on exploring the relationship between apical healing status after root canal treatment (RCT) and DM, or on narrative reviews regarding DM and AP. This systematic review aims to analyze the association between PGC and AP in diabetic patients, with a particular emphasis on the clinical implications that may be involved.

However, previous systematic reviews have been limited by small sample sizes, restricted time frames, and a lack of recent evidence published after 2020. Moreover, earlier reviews have seldom distinguished between the prevalence of AP and the persistence of apical periodontitis in root-filled teeth (AP-RFT), and none have integrated findings from immunological or microbiological studies into the overall interpretation.

Therefore, this review seeks to provide an updated and more comprehensive synthesis by incorporating newly published studies, separately evaluating AP and AP-RFT outcomes, and summarizing emerging immune-inflammatory mechanisms that may underlie the relationship between glycemic control and periapical disease.

## Material and Methods

The protocol for this systematic review was developed in accordance with the PRISMA 2020 guidelines (25). The protocol of this review has been registered in the PROSPERO database under registration number CRD420251078354.

Focused Question and Eligibility Criteria

This review aims to evaluate whether PGC in diabetic patients is associated with the prevalence of AP, the persistence or recurrence after RCT, and alterations in local or systemic immune-inflammatory responses. Given the inconsistent findings reported in studies over the past two decades, we sought to systematically review and synthesize evidence published within the last 20 years.

Because PGC is evaluated as an exposure rather than an intentional intervention, the PECO framework was adopted. Unlike PICO, which is suited for assessing predefined interventions in experimental studies, PECO more appropriately reflects the observational nature of PGC and the designs of the included studies, providing a conceptually accurate structure for defining exposure, comparison groups, and clinical outcomes.

Based on the PECO framework, the components of the research question are defined as follows:

- P (Population): Diabetic patients.

- E (Exposure): PGC (HbA1c &gt; 6.5%, 7%, or 8%, etc.).

- C (Comparison): Diabetic patients with good glycemic control (GGC) or non-diabetic individuals.

- O (Outcome):

1. Prevalence of AP.

2. Persistence of AP-RFT.

3. Local or systemic immune-inflammatory responses related to AP.

Based on this structure, the following focused questions were developed:

1. Does PGC increase the prevalence of AP or impair its healing following RCT?

2. Is PGC associated with alterations in local or systemic immune-inflammatory responses in patients with AP?

The study applied the following inclusion and exclusion criteria, which were designed to ensure the selection of relevant and methodologically appropriate studies while excluding those that did not meet the predefined requirements.

The inclusion criteria were as follows: Articles published in English; study types including cross-sectional studies, case-control studies, cohort studies, and longitudinal studies involving human participants; experimental groups must involve diabetic patients with PGC, and the control group may be diabetic patients with GGC or non-diabetic individuals. The glycemic control level must be clearly defined using HbA1c values (6.5%, 7%, or 8%); and outcome measures should include at least one of the following: The prevalence of AP, the persistence of AP-RFT, or immune response, with the first two assessed through radiographic examination.

Exclusion criteria included the following: Animal studies, case reports, conference abstracts, and literature reviews; studies that only include diabetic patients without a control group, or studies with a control group that do not specify the stratification of participants based on HbA1c values; studies involving participants under 18 years of age; and studies with a sample size smaller than 15 participants.

Study Selection

The electronic databases for the literature search include PubMed, Embase, Google Scholar, and Scopus. The search period ranges from July 2005 to June 2025. Keywords used in the search include: "apical periodontitis", "diabetes", "glycemic control", "glycated hemoglobin", "root canal treatment" and their synonyms, combined using Boolean operators "AND" and "OR" to optimize the search results. The full and detailed search strategies for each database are provided in Supplementary Table S1 (http://www.medicina.oral.com/carpeta/suppl1_27997).

Two independent researchers (JL and XW) conducted the literature search in June 2025. First, they collected and screened the titles and abstracts of the publicly available articles. After excluding obviously irrelevant studies, they proceeded to full-text screening. Inter-reviewer agreement was assessed using Cohen's kappa (), which demonstrated substantial agreement for title and abstract screening (=0.70) and excellent agreement for full-text assessment (=0.85). Data were summarized using a standardized data extraction form, and final inclusion decisions were made through consensus, with consultation from a third reviewer (SG) in case of disagreements. The extracted information included the following: Author names, publication year, study design, study groupings, sample size, odds ratio (OR), and P-value.

Quality Assessment

Each included study was independently reviewed by two assessors using the Joanna Briggs Institute (JBI) Critical Appraisal Tool (26) to assess the risk of bias. The methodological quality of each study was independently evaluated to determine how well potential biases were addressed in the design, implementation, and analysis. The following eight aspects were considered: Were the inclusion criteria clearly defined?; were the study subjects and setting adequately described?; was the exposure measured reliably and validly?; were objective and standard criteria used for disease measurement?; were confounding factors identified?; were strategies for addressing confounding factors stated?; were outcomes measured reliably and validly?; and was the statistical analysis appropriate and reasonable?

Outcome Variables and Statistical Analysis

The outcome variables were categorized into three main areas: The impact of PGC on the prevalence of AP; the impact of PGC on the persistence of AP-RFT; and the effect of PGC on local or systemic immune responses in DM patients with AP.

For the first two major categories of analysis, dichotomous meta-analyses were performed to calculate pooled odds ratios (ORs) with corresponding 95% confidence intervals (CIs). The analyzed variables included the prevalence of AP and the persistence of AP-RFT among diabetic patients with PGC. The ORs for these two outcomes were visualized using forest plots, and heterogeneity was assessed using Chi-square and I² tests, with the significance level set at P<0.05. The I2 value was used to assess the extent of heterogeneity: If I2&gt;50%, a random-effects model was used; otherwise, a fixed-effects model was applied. RevMan 5.3 software (The Nordic Cochrane Centre, The Cochrane Collaboration, Copenhagen, 2014) was used for the meta-analysis. For the third category (immune responses), due to the inherent heterogeneity of the outcome data, a narrative synthesis approach was adopted.

## Results

Study Selection

**Figure 1:**
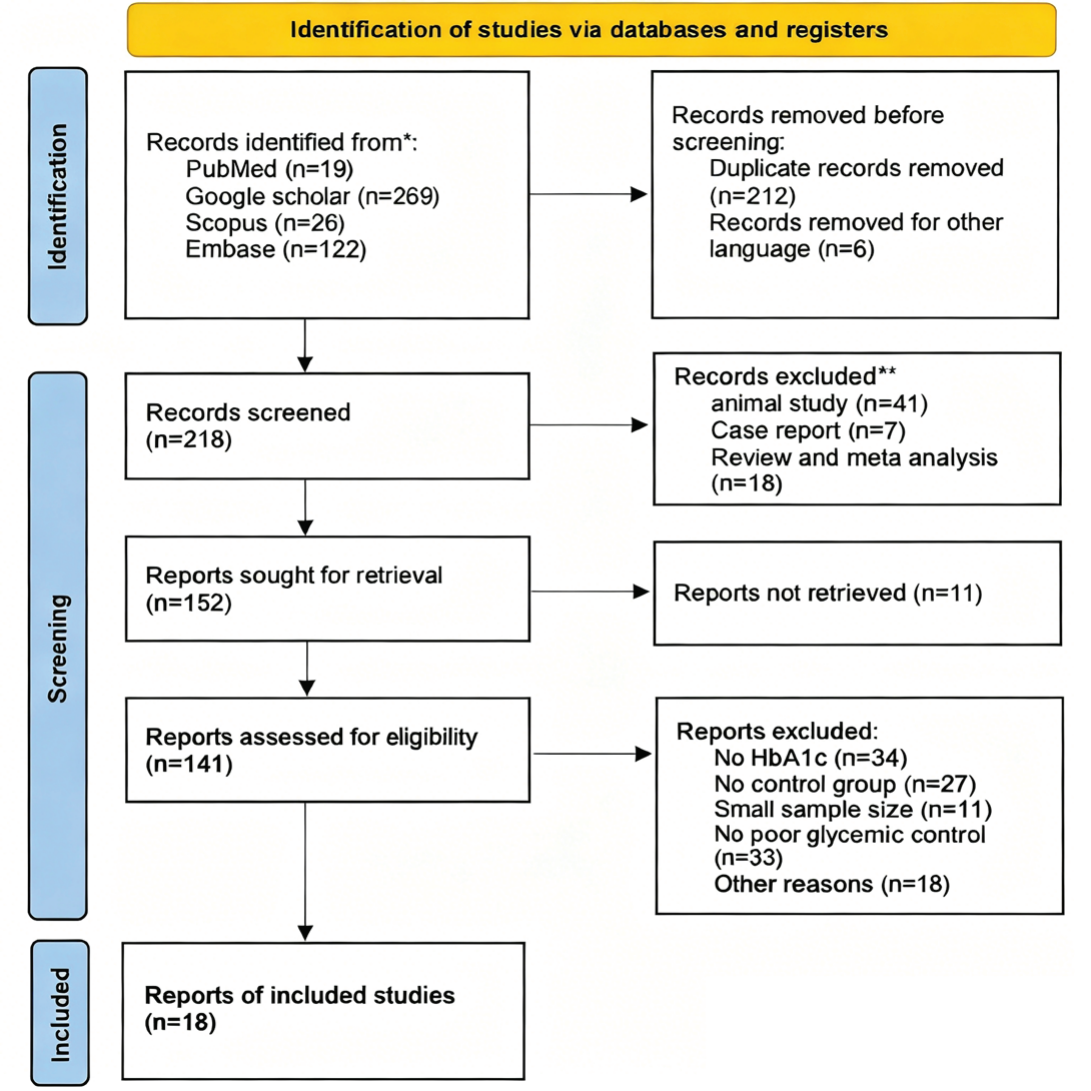
Adapted PRISMA 2020 flow diagram showing the selection process of included studies. This diagram summarizes the identification, screening, eligibility assessment, and final inclusion of studies following the PRISMA 2020 guidelines. Reasons for exclusion at each stage are provided, including removal of duplicates, exclusion of non-English publications, ineligible study types, small sample size, and lack of relevant glycemic or outcome data.

A total of 436 potentially relevant articles were identified through electronic database searches and manual screening. After removing 212 duplicate records and excluding 6 non-English publications, the remaining articles were screened based on the predefined inclusion and exclusion criteria. Following full-text assessment, 18 studies (27 - 44) were ultimately included in the systematic review and meta-analysis. The detailed study selection process is illustrated in Figure 1.

1

General Characteristics of Included Studies

The general characteristics of the 18 included studies (27 - 44) are summarized in Table 1.

Table 1

Among them, four were prospective cohort studies (27 - 30), and the remaining fourteen were cross-sectional studies (31 - 44). In the prospective studies, three (27 - 29) employed non-surgical root canal treatment (NSRCT) as an intervention and included a one-year follow-up period, while one study (30) evaluated the two-year tooth extraction rate following NSRCT. Among the cross-sectional studies, five (31 - 35) investigated a variety of biological and microbiological markers, including immune-related biomarkers, proteins, bacteria, and endotoxins. Two studies (37 , 41) assessed only the prevalence of AP based on radiographic examination. One study (39) reported solely the Persistence of AP-RFT. Five studies (36 , 38 , 40 , 42 , 43) assessed the prevalence of both AP and AP-RFT. One study (44) reported both the prevalence of AP and the levels of bacteria and endotoxins.

Among the 15 studies that reported the prevalence of AP or AP-RFT, ten (28 - 29 , 36 - 40 , 42 - 44) utilized the periapical index (PAI) based on either conventional radiographs or cone-beam computed tomography (CBCT); one study (27) applied the Strindberg criteria; one (41) did not specify the assessment criteria; and one study (30) did not involve any radiographic evaluation.

Collectively, these studies involved a total of 51,070 participants. Glycemic control in diabetic patients was consistently categorized based on HbA1c levels. Twelve studies (28 , 30 , 31 , 33 - 37 , 39 - 40 , 42 , 44) used a threshold of approximately 6.5%; five studies (27 , 29 , 32 , 38 , 41) used thresholds ranging from 7.0% to 7.5%; and one study (43) used a value around 8.0%. Regarding unit of analysis, two studies (30 , 39) conducted analyses at the tooth level, while the remaining sixteen (27 - 29 , 31 - 38 , 40 - 44) analyzed data at the individual level. In terms of outcomes, one study (41) reported that good glycemic control was paradoxically associated with an increased prevalence of AP; three studies (31 , 34 , 40) found no significant association between glycemic control and AP; and the remaining fourteen studies (27 - 30 , 32 - 33 , 35 - 39 , 42 - 44) identified either a significant or a potential association between poor glycemic control and the presence of AP.

Methodological Quality Assessment

A total of 18 studies were evaluated using the JBI critical appraisal checklists for cross-sectional and Prospective cohort study designs. Five studies met the criteria for high methodological quality, while the remaining thirteen were judged to be of moderate quality; no studies were considered low quality (Table 2).

Table 2

Overall, the main methodological limitations were related to the inadequate identification of confounding factors, lack of strategies to address potential confounders, and the absence of regression modeling or adjustment for confounding variables in statistical analyses.

Meta-analysis

Association Between PGC and the Prevalence of AP

A total of eight studies (36 - 38 , 40 - 44) employed cross-sectional designs using radiographic methods to investigate whether PGC is associated with the prevalence of AP. Among them, the studies by Silva (41) and Slceanu (43) did not report the raw prevalence data for the case and control groups. Instead, they presented outcomes using mean differences (MD) and standard deviations (SD), which are considered continuous variables and thus not suitable for inclusion in this binary outcome meta-analysis.

**Figure 2:**
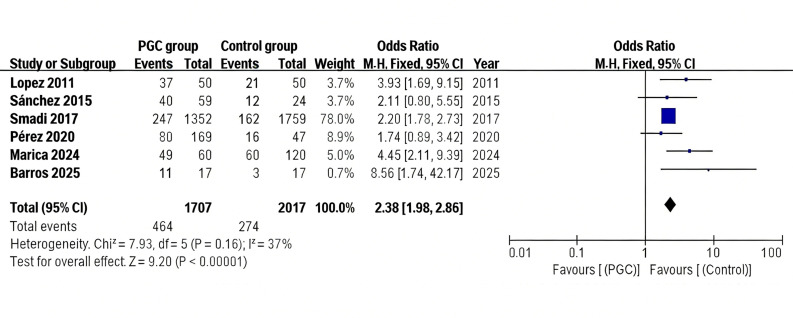
Forest plot of ORs for PGC and prevalence of AP. This figure presents individual study estimates and the pooled odds ratio (OR) comparing poor glycemic control (PGC) with non-PGC groups for the prevalence of apical periodontitis (AP). A fixed-effects model, 95% confidence intervals (CIs), study weights, and overall heterogeneity statistics (I²) are shown.

After excluding these two studies, six studies (36 - 38 , 40 , 42) were included in the final analysis. A forest plot was generated to present the odds ratios (ORs) from individual studies as well as the pooled OR derived from the meta-analysis (Figure 2). The pooled OR was 2.38 (95% CI=1.98-2.86; ²=7.93; P<0.00001). As the heterogeneity among studies was low (I²=37%), a fixed-effects model was applied. These results indicate that PGC in diabetic patients is significantly associated with an increased prevalence of AP.

2

Association Between PGC and the Persistence of AP-RFT

A total of 10 studies (27 - 30 , 36 , 38 - 40 , 42 - 43) employing either cross-sectional or longitudinal designs, investigated the association between PGC and the persistence or healing outcome of AP-RFT. Among them, the study by Slceanu et al. (43) was excluded due to the use of continuous outcomes, which were incompatible with the dichotomous meta-analysis format; the study by Wang et al. (30) was excluded due to substantial outcome heterogeneity in outcome measures, as it only reported the extraction rate following NSRCT rather than providing data on the persistence of AP-RFT.

**Figure 3:**
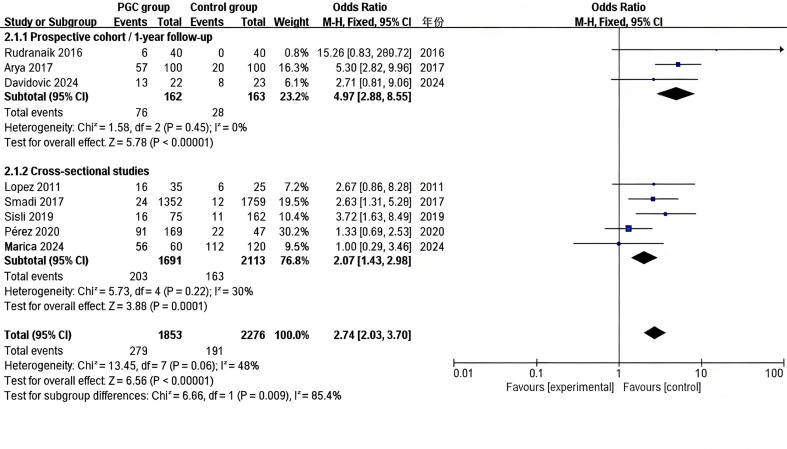
Forest plot of ORs for PGC and persistence of AP-RFT. This figure displays the association between poor glycemic control (PGC) and the persistence of apical periodontitis in root-filled teeth (AP-RFT). Subgroup analyses illustrate differences in effect estimates between prospective cohort studies with a standardized 1-year follow-up and cross-sectional studies, with subgroup-specific pooled ORs, 95% CIs, heterogeneity measures (I²), and the test for subgroup differences.

After excluding these two studies, eight studies (27 - 29 , 36 , 38 - 40 , 42) were included in the final analysis. A forest plot was used to illustrate the ORs from each study and the pooled OR from the meta-analysis (Figure 3).

3

The overall result indicated a significantly increased risk of AP-RFT in patients with PGC, with a pooled OR of 2.74 (95% CI: 2.03-3.70; ²=13.45; P<0.00001). Given the moderate level of heterogeneity (I²=48%), a fixed-effects model was applied. To explore the heterogeneity observed in the primary analysis (I²=48%), subgroup analyses were conducted based on study design and follow-up duration. Studies with a prospective design and a standardized 1-year follow-up (n=3) demonstrated a consistently higher risk estimate, with a pooled OR of 4.97 (95% CI: 2.88-8.55;I²=0%), indicating that the association between PGC and persistent AP-RFT was stronger and more homogeneous when follow-up periods were clearly defined. In contrast, cross-sectional studies (n=5) showed a weaker but still significant association (pooled OR=2.07; 95% CI: 1.43-2.98), accompanied by greater heterogeneity I²=30%), suggesting that differences in assessment timing across cross-sectional designs may contribute to the overall variability. The significant subgroup difference (P=0.009) further indicates that study design and follow-up duration are key contributors to the observed heterogeneity. These findings suggest that PGC in diabetic patients is significantly associated with an increased incidence of AP-RFT.

Sensitivity Analysis

To assess the robustness of the meta-analysis results, a leave-one-out sensitivity analysis was performed. This involved sequentially removing each included study and recalculating the pooled effect estimate. The results indicated that exclusion of any single study did not substantially alter the overall effect size, suggesting that the findings are stable and robust. Given that only 6 to 8 studies were ultimately included in the meta-analysis, the number of studies did not meet the commonly recommended threshold (n10) for conducting a funnel plot analysis. Therefore, publication bias was not assessed in this study. Future analyses incorporating a larger number of studies may allow for a more comprehensive evaluation of potential publication bias.

Narrative Synthesis of Local and Systemic Immune Responses

This section includes six studies aimed at investigating the potential impact of glycemic control on local and systemic immune responses in patients with AP (31 - 35 , 44). Due to substantial heterogeneity in outcome measures, assessment methods, and reporting formats, a meta-analysis was not performed. Instead, a qualitative narrative synthesis was conducted. The key immune-inflammatory, proteomic, and microbiological findings from these studies are summarized in Table 3.

Table 3

All included studies were cross-sectional in design, comparing T2DM patients with PGC to non-diabetic controls. Biological samples analyzed included periapical lesion tissues, root canal contents, and peripheral blood. The employed analytical methods encompassed immunohistochemistry, proteomic profiling, and microbiological analysis. The sample sizes ranged from 18 to 280, with units reported as either patients or teeth depending on study design.

Regarding inflammatory and immune-related markers, Sarmento et al. (31) first evaluated the expression of matrix metalloproteinase-9 (MMP-9), Receptor activator of nuclear factor B ligand (RANKL), and parathyroid hormone-related protein (PTHrP) in periapical tissues in 2020, and found no statistically significant differences between diabetic and non-diabetic groups. In a subsequent study published in 2023, the same group reported a significantly elevated expression of interleukin-17 (IL-17) in T2DM patients with PGC (P=0.047), while interleukin-1 (IL-1) and tumor necrosis factor-alpha (TNF-) remained statistically non-significant (33). Dhamija et al. (35) observed significantly elevated levels of interleukin-6 (IL-6), TNF-, and high-sensitivity C-reactive protein (hsCRP) in poorly controlled T2DM patients with AP (P<0.01), while IL-1 levels did not differ significantly. In contrast, Aldoss et al. (34) compared IL-17 expression among T2DM patients with PGC, those with GGC, and normoglycemic individuals, and found no statistically significant differences among the three groups (P=0.281).

In the field of proteomics, Loureiro et al. (32) identified 43 upregulated and 22 downregulated proteins in root canal samples from T2DM patients with PGC (P<0.05), while the expression of 59 proteins showed no significant differences (P&gt;0.05). These findings suggest that glycemic status may influence the protein expression profile within the endodontic microenvironment. However, the specific biological functions and clinical relevance of these proteins warrant further investigation.

As for microbiological analysis, Aldoss et al. (34) found no significant differences in intraradicular bacterial load among T2DM patients with PGC, those with GGC, and normoglycemic individuals (P=0.613), suggesting that bacterial quantity may not be the primary factor influencing disease status, and that host immune response could play a more pivotal role in disease progression. Conversely, Barros et al. (44) reported significantly elevated bacterial loads and endotoxin levels in T2DM patients with PGC (P<0.05), indicating that hyperglycemia may be associated with increased microbial virulence or intensified host-pathogen interactions.

In summary, current evidence suggests that PGC may be associated with elevated levels of certain pro-inflammatory cytokines (e.g., IL-6, TNF-, IL-17), as well as distinct patterns in protein expression and microbial indicators. However, notable variations in sample types, analytical techniques, and grouping criteria across studies limit the comparability and consistency of findings. Accordingly, quantitative synthesis was not feasible for this outcome. Further high-quality, mechanism-focused research is needed to elucidate the immunopathological links between hyperglycemia and AP.

## Discussion

Current Research Landscape The potential association between DM and AP has only gained attention in the past two decades, with systematic reviews on this topic primarily emerging within the last ten years. To date, approximately six systematic reviews and meta-analyses (45 - 50) have investigated the relationship between DM and periapical healing outcomes following RCT. However, only one review (51) has specifically addressed the prevalence of AP in patients with diabetes. Although the number of included studies was limited, the existing evidence consistently supported a positive association between glycemic dysregulation and AP. Notably, there is a lack of systematic reviews that comprehensively explore the pathophysiological link between glycemic control and AP from the perspective of immune-inflammatory mechanisms, which remains an underexplored area in the current literature. With the progression of this line of research, several review articles have proposed analyzing the relationship between AP and metabolic dysregulation associated with hyperglycemia (52 - 53). These study designs encompassed longitudinal, cross-sectional, and animal experiments. For instance, in a one-year longitudinal study, Davidovi et al. (29) reported periapical healing rates of 79.2% in non-diabetic patients, 65.2% in diabetic patients with GGC, and only 40.9% in those with PGC following RCT. The intergroup analysis further revealed a statistically significant difference in treatment success rates between PGC and non-diabetic groups (P=0.008). Similarly, a large cross-sectional study by Yip et al. (54) demonstrated a significant association between PGC, as reflected by elevated HbA1c levels, and the prevalence of AP. Using a diabetic mouse model, Cintra et al. (55) found that apical infection not only elevated blood glucose levels in diabetic mice but also increased HbA1c levels in both diabetic and non-diabetic subjects, suggesting a possible bidirectional link between AP and metabolic imbalance. This systematic review and meta-analysis aimed to investigate whether poorly controlled hyperglycemia is significantly associated with AP. By examining the occurrence, prognosis, and immune-microbial characteristics of AP in diabetic patients with PGC, we sought to provide a deeper understanding of the multifaceted link between these two chronic conditions and to encourage future research into their underlying immunopathological mechanisms. Summary of Meta-analysis and Key Findings on Immune Mechanisms Among the 18 studies (27 - 44) included in this review, 13 studies (27 - 30 , 36 - 44) investigated the association between glycemic control status and the prevalence of AP, the prevalence of AP-RFT, or both. Specifically, eight studies (36 - 38 , 40 - 44) assessed the association between HbA1c levels and the prevalence of AP, while ten studies (27 - 30 , 36 , 38 - 40 , 42 - 43) focused on the risk of recurrence of AP-RFT. First, among the eight studies analyzing the prevalence of AP (36 - 38 , 40 - 44), six (36 - 38 , 42 - 44) reported a significant association between PGC and an increased risk of AP. One study (40) found no significant correlation, while another (41) observed a higher prevalence of AP in patients with GGC. The authors of the latter speculated that this result may be attributable to the shorter duration of metabolic disturbance in the GGC group, which may not have been sufficient to induce irreversible periapical changes. The meta-analysis results indicated that individuals with PGC had a significantly higher risk of developing AP compared to controls (P<0.00001). Second, among the ten studies addressing AP-RFT (27 - 30 , 36 , 38 - 40 , 42 - 43), seven (27 - 30 , 38 , 39 , 43) demonstrated a significant association between PGC and increased risk of recurrence following RCT, whereas three studies (36 , 40 , 42) did not report significant differences. The pooled meta-analysis similarly revealed that, despite undergoing endodontic therapy, patients with PGC exhibited a significantly higher Persistence of AP-RFT (P<0.00001). In addition, six studies (31 - 35 , 44) explored the potential links between glycemic control status and the immune and microbial profiles in patients with AP. Among them, two studies (35 , 44) reported significantly elevated bacterial loads and increased expression of proinflammatory cytokines (IL-6 and TNF-) in patients with PGC. However, these findings were inconsistent with those of two other studies (33 , 34), which did not observe significant differences in microbial or cytokine levels. Notably, Sarmento et al. (33) reported a significant upregulation of IL-17 expression, further emphasizing the complexity and heterogeneity of immune-inflammatory responses associated with glycemic status.Proteomic profiling by Lopes et al. (32) identified 43 upregulated and 22 downregulated proteins in patients with PGC, implicated in immune modulation, bone remodeling, and oxidative stress pathways. In contrast, IL-1, RANK, MMP-9, and a majority of the remaining 59 proteins did not show statistically significant changes(31 - 33 , 35). Collectively, these findings suggest that hyperglycemic conditions may contribute to the development and progression of AP, at least in part, through proinflammatory immune pathways. Nevertheless, considerable heterogeneity persists across studies in terms of sampling methods, detection technologies, and outcome measures. Particularly in proteomic studies, although several candidate molecular pathways have been proposed, their biological significance and clinical implications remain to be validated in large-scale investigations employing standardized protocols. Sources and Interpretation of Heterogeneity This study adhered strictly to the PECO framework, which is appropriate for observational study designs, for study selection and meta-analysis. All included articles were published between 2011 and 2025, which helps to reduce the risk of temporal bias associated with evolving standards in RCT protocols or diagnostic criteria for DM. Nevertheless, a certain degree of heterogeneity was observed across the included studies. Apart from the differences in study design and follow-up duration identified in the subgroup analysis, heterogeneity was mainly reflected in the following four areas: First, heterogeneity in analytical units and inclusion criteria was evident. While most studies used individual patients as the unit of analysis, the study by Wang et al. (30) analyzed 49,334 teeth, each from a different patient. Despite this, the use of teeth rather than patients as the unit of analysis introduced a methodological discrepancy compared to other studies. In contrast, Sisli et al. (39) analyzed 237 teeth derived from only 99 patients, indicating that multiple teeth from the same individual were included, which may compromise the assumption of statistical independence. Second, differences were found in group stratification and definitions of exposure. Although most studies used non-diabetic individuals as the control group, some, such as those by Sánchez et al. (37), Pérez et al. (40), and Silva et al. (41), used diabetic patients with GGC as controls. Smadi et al. (38) and Sisli et al. (39) further stratified their participants into three groups: PGC, GGC, and non-diabetics. Moreover, the threshold for defining PGC based on HbA1c levels varied among studies. While most adopted a cutoff of 6.5%, some set it between 7.0% and 7.5%, and in the study by Slceanu et al. (43), the average HbA1c level in the experimental group was approximately 8.0%. Although the threshold definitions were based on different clinical guidelines, such variations in exposure definitions may have affected the comparability and homogeneity of results. Third, outcome assessment methods varied substantially. Imaging modalities used to assess AP varied and included periapical radiographs, panoramic radiographs, and CBCT. Diagnostic criteria for AP were also inconsistent: Most studies used PAI3 as the threshold, while Slceanu et al. (43) defined AP as PAI=1. Finally, studies incorporating immunological, inflammatory, or proteomic biomarkers differed in both target selection and detection methods, contributing to additional inter-study heterogeneity. Despite these methodological and measurement discrepancies, the meta-analysis consistently demonstrated a statistically significant association between PGC and both the clinical incidence and post-treatment recurrence of AP. These findings suggest a certain level of robustness in the overall conclusion. However, the interpretation should be undertaken with caution and carefully contextualized in light of the specific study designs and methodological characteristics of the included studies. Mechanistic Insights and Clinical Implications As early as 1963, Professor Bender proposed that pulp arteritis in diabetic patients might cause toothache without carious lesions, introducing an early hypothesis linking DM and dental disease (56). The interplay between hyperglycemia and AP has been explored through several plausible biological mechanisms (57). Chronic hyperglycemia induces microvascular dysfunction, reduces tissue oxygenation, and impairs both neutrophil and macrophage activity, thereby creating a permissive microenvironment for infection and delaying periapical healing. Diabetes-related alterations-such as diminished neutrophil chemotaxis and enhanced production of reactive oxygen species-predispose patients with PGC to heightened inflammatory activity and impaired bacterial clearance. At the molecular level, hyperglycemia has been shown to upregulate proinflammatory cytokines, including IL-1, IL-6, and TNF-, and to enhance osteoclastic activity through dysregulation of the RANKL/OPG axis (58 - 59). Impaired immune function further amplifies inflammation and oxidative stress, contributing to bone resorption and delayed repair of periapical tissues (60 - 61). These mechanisms align with the immune-inflammatory findings summarized in this review and offer a biological basis for the observed associations between PGC and both the development and persistence of AP. From a clinical perspective, these mechanistic insights underscore the importance of integrating glycemic assessment into the dental management of diabetic patients. Screening for unrecognized hyperglycemia-or identifying PGC in individuals presenting with recurrent infections, persistent periapical lesions, or delayed healing-may enhance diagnostic accuracy and risk stratification. Preoperative evaluation of HbA1c levels can assist clinicians in anticipating complications and tailoring treatment plans, including consideration of enhanced disinfection protocols, supplementary recall visits, or more cautious prognostic counseling. Moreover, diabetic patients-particularly those with PGC-may benefit from shortened recall intervals to monitor healing and to detect early signs of persistent AP-RFT. Interdisciplinary collaboration between endodontists and endocrinologists is essential to ensure optimal systemic and oral health outcomes. As evidence continues to accumulate regarding the bidirectional relationship between metabolic status and endodontic disease, personalized treatment strategies that account for glycemic control will become increasingly important in routine clinical practice. Limitations and Future Directions This systematic review and meta-analysis has several limitations that should be considered when interpreting the findings. First, despite being published within the last 20 years, the included studies exhibited notable methodological heterogeneity, including differences in study design, analytical units, exposure definitions, imaging modalities, and diagnostic criteria for AP. Such variability may have affected the comparability and consistency of outcome assessments. Second, the overall quality of evidence was constrained by variation in sample size and the predominance of cross-sectional study designs, which limit causal inference and reduce the robustness of pooled estimates. Third, although several studies investigated immunological, inflammatory, or proteomic markers, the diversity in sampling protocols, biomarker panels, and detection platforms hindered the ability to synthesize mechanistic pathways. Fourth, potential confounders-including RCT quality, duration of diabetes, systemic comorbidities, and medication use-were inconsistently reported and insufficiently controlled for, which may have introduced residual confounding. Lastly, the number of studies included in each meta-analysis was relatively small, thereby limiting the statistical power and precision of the pooled estimates. Because all eligible evidence was derived from observational studies-most of them cross-sectional-causality cannot be inferred from the associations identified between glycemic control and AP or AP-RFT. The findings should therefore be interpreted as correlational rather than causal. Future research should prioritize well-designed, adequately powered prospective studies employing standardized criteria for glycemic assessment, AP diagnosis, and follow-up duration. Multicenter studies using uniform imaging modalities and diagnostic thresholds would greatly improve comparability and enhance the reliability of pooled estimates. In addition, integrating microbiological, immunological, and proteomic data with clinical and radiographic outcomes may yield deeper insights into the biological mechanisms linking hyperglycemia and periapical pathology. Longitudinal studies examining whether improvements in glycemic control translate into enhanced periapical healing or reduced recurrence rates represent a particularly promising direction. Close collaboration between endodontists and endocrinologists will remain essential to optimize both systemic and dental outcomes in patients with diabetes. This review adds value beyond previous meta-analyses by incorporating newly published studies, distinguishing between disease prevalence and post-treatment persistence, and synthesizing emerging evidence on immune and microbial pathways associated with glycemic dysregulation. These contributions provide a more contemporary and mechanistically informed understanding of how poor glycemic control may influence periapical disease, thereby offering updated insights for both future research and clinical practice.

## Conclusions

This systematic review and meta-analysis indicate that patients with diabetes-particularly those with PGC-have a significantly increased risk of developing AP. Moreover, diabetes may adversely affect the prognosis of RCT and long-term tooth retention. Despite some heterogeneity among studies, the overall evidence supports a clear association between DM and AP. Dysregulated immune responses, activation of inflammatory mediators, and alterations in the oral microbiome are likely key mechanisms through which hyperglycemia contributes to the onset and progression of periapical disease. These findings not only enhance our understanding of the underlying pathophysiology but also offer novel therapeutic targets for clinical intervention. Given the existing variability in study design, outcome definitions, and data standardization, future research should focus on high-quality, mechanism-driven prospective studies. In particular, the integration of proteomics, dynamic monitoring of inflammatory biomarkers, and interventional studies will be crucial for elucidating the causal relationship and developing personalized management strategies for high-risk populations.

## Figures and Tables

**Table 1:** Table General characteristics of included studies.

NO.	Study (Author, Year)	Study Design / Follow-up Duration	Sample Type and Methods	Grouping(T vs. C)	Sample Size (n, T vs. C)	Main Outcome Indicators	Association and Key Findings
1	Rudranaik,2016 [27]	Prospective cohort / 1 year	NSRCT	T2DM (HbA1c>7.5%)vs. Non-DM	80 patients (40 vs. 40)	Persistence of AP-RFT (Strindberg Criteria) after NSRCT	Yes, higher AP-RFT in T2DM (HbA1c>7.5%) (P=0.02).
2	Arya,2017 [28]	Prospective cohort / 1 year	NSRCT	T2DM (HbA1c>6.5%)vs. Non-DM	46 patients (21 vs. 25)	Persistence of AP-RFT (PAI>3) after NSRCT	Yes, higher AP-RFT in T2DM (HbA1c>6.5%) (P<0.05).
3	Davidović,2024 [29]	Prospective cohort / 1 year	NSRCT	T2DM (HbA1c >= 7% and HbA1c < 7%) vs. Non-DM patients	75 patients (25+25 vs. 25)	Persistence of AP-RFT (PAI>3) after NSRCT	Yes, higher AP-RFT in T2DM (HbA1c>=7%) (P=0.022).
4	Wang,2011 [30]	Prospective cohort / 2 years	HbA1c data collection and longitudinal tooth follow-up	T2DM (HbA1c>=6.5%) vs. Non-DM	49,334 teeth (4,358 vs. 44,976)	HbA1c values; Tooth extraction rate after NSRCT (Radiographic assessment not metioned)	Yes, higher AP-RFT-related extraction rate in T2DM (HbA1c >=6.5%) (P=0.0008).
5	Sarmento,2020 [31]	Cross-sectional	Periapical tissue sampling and IHC analysis	T2DM (HbA1c>6.4%) vs. Non-DM	26 patients (13 vs. 13)	PTHrP, RANKL, MMP-9 expression in periapical tissues	No, RANKL (P=0.26); MMP-9 (P=0.17); PTHrP (P=0.43).
6	Loureiro,2022 [32]	Cross-sectional	Root canal sampling and proteomic analysis	T2DM (HbA1c>=7.5%) vs. Non-DM	18 patients (9 vs. 9)	124 kinds of proteins	(1) Yes, 43 upregulated (P<0.05); 22 downregulated (P<0.05).(2) No, 59 proteins (P>0.05).
7	Sarmento,2023 [33]	Cross-sectional	Periapical tissue sampling and IHC analysis	T2DM (HbA1c>6.4%) vs. Non-DM (HbA1c<5.7%)	26 patients (13 vs. 13)	IL-17, IL-1Î², TNF-Î± expression in apical periapical lesions	(1) Yes, IL-17 (P=0.047).(2) No, IL-1Î² (P>0.05); TNF-Î± (P>0.05).
8	Aldoss,2023 [34]	Cross-sectional	Root canal sampling, bacterial and IHC analysis	T2DM (HbA1c>=6.5% and HbA1c= 5.7-6.4%) vs. Non-DM (HbA1c<5.7%)	65 patients (20+23 vs. 22)	Bacterial count; IL-17 expression in canal	(1) No, bacterial counts (P=0.613);(2) No, IL-17 (P=0.281).
9	Dhamija,2025 [35]	Cross-sectional	Peripheral blood sampling and IHC analysis	T2DM and T2DM-AP (HbA1c>6.5%) vs. Non-DM and Non-DM-AP	280 patients (70+70 vs. 70+70)	IL-1Î², IL-6, TNF-Î± hsCRP, HbA1c	(1) Yes, IL-6 (P<0.01); TNF-Î± (P<0.01); hsCRP (P<0.01).(2) No, IL-1Î² (P>0.05).
10	Lopez,2011 [36]	Cross-sectional	Radiographic examination (Panoramic)	T2DM (HbA1c=6.6±0.6%) vs. Non-DM	100 patients (50 vs. 50)	Prevalence of AP and RFT-AP (PAI>=3)	(1) Yes, higher AP in T2DM (HbA1c=6.6±0.6%) (P<0.01).(2) No, AP-RFT (P>0.05).
11	SÃ¡nchez,2015 [37]	Cross-sectional	Radiographic examination (Panoramic)	T2DM (HbA1c>=6.5%) vs. T2DM (HbA1c<6.5%)	83 patients (59 vs. 24)	Prevalence of AP (PAI>=3);	Yes, higher AP in T2DM (HbA1c>=6.5%) (P=0.03).
12	Smadi,2017 [38]	Cross-sectional	Radiographic examination (Panoramic)	T2DM (HbA1c>=7% and HbA1c<7%) vs. Non-DM	291 patients (63+82 vs. 146)	Prevalence of AP and RFT-AP (PAI>=3)	Yes, higher AP and AP-RFT in T2DM (HbA1c>=7%) (P<0.05);
13	Sisli,2019 [39]	Cross-sectional	Radiographic examination (CBCT)	T2DM (HbA1c>=6.5% and HbA1c<6.5%) vs. Non-DM	237 teeth (40+35 vs. 162)	Persistence of RFT-AP (CBCTPAI>=3)	(1) Yes, higher AP-RFT in T2DM (P<0.05).(2) No, RFT-AP between T2DM (HbA1c>=6.5%) and T2DM (HbA1c<6.5%) (P>0.05).
14	PÃ©rez,2020 [40]	Cross-sectional	Radiographic examination (Panoramic)	T2DM (HbA1c>=6.5%) vs. T2DM (HbA1c<6.5%)	216 patients (169 vs. 47)	Prevalence of AP and RFT-AP (PAI>=3)	No, AP (P>0.05); AP-RFT (P>0.05).
15	Silva,2021 [41]	Cross-sectional	Radiographic examination (Periapical)	T2DM (HbA1c>=7%) vs. T2DM (HbA1c<7%)	27 patients (16 vs. 11)	Prevalence of AP (Diagnostic criteria not specified)	Yes, higher AP in T2DM (HbA1c<7%) (P<0.05).
16	Marica,2024 [42]	Cross-sectional	Radiographic examination (Panoramic)	T2DM (HbA1c>6.5%) vs. Non-DM	180 patients (60 vs. 120)	Prevalence of AP and RFT-AP (PAI>=3)	(1) Yes, higher AP in T2DM (HbA1c>6.5%) (P<0.001).(2) No, AP-RFT (P>0.05).
17	Sălceanu,2025 [43]	Cross-sectional	Radiographic examination (Panoramic)	T2DM (HbA1c=8±1.7 %) vs. Non-DM	90 patients (55 vs. 35)	Prevalence of AP and RFT-AP (PAI>=1);	Yes, higher AP and AP-RFT in T2DM (HbA1c>=7%) (P<=0.01);
18	Barros,2025 [44]	Cross-sectional	Root canal sampling, bacterial and endotoxin analysis, radiographic examination (CBCT)	T2DM (HbA1c>=6.5%) vs. Non-DM	34 patients (17 vs. 17)	Prevalence of AP (CBCTPAI>=4); bacterial load; endotoxin levels.	Yes, higher AP in T2DM (HbA1c>=6.5%) (P<0.05); bacterial load (P<0.05); endotoxin levels (P<0.05).

T: Test group; C: Control group; NSRCT: Nonsurgical root canal treatment; T2DM: Type 2 diabetic mellitus; HbA1c: Glycated haemoglobin; Non-DM: Nondiabetic mellitus; AP: Apical periodontitis; AP-RFT: Apical periodontitis in root-filled teeth; PAI: Periapical index; IHC: Immunohistochemical; PTHrP: Parathyroid hormone-related protein; RANKL: Receptor activator of nuclear factor kappa-B ligand; MMP-9: Matrix metalloproteinase-9; IL-17: Interleukin-17; IL-1β: Interleukin-1 beta; TNF-α: Tumor necrosis factor-alpha; IL-6: Interleukin-6; hsCRP: High-sensitivity C-reactive protein; CBCT: Cone-beam computed tomography.

**Table 2:** Table Methodological quality for cross-sectional studies.

NO.	Author(Year)	Q1	Q2	Q3	Q4	Q5	Q6	Q7	Q8	Total Score (n/16)	Level of Quality
1	Wang, 2011 [30]	Yes	Yes	Yes	UC	Yes	Yes	Yes	Yes	15/16	High
2	Lopez, 2011 [36]	Yes	Yes	Yes	Yes	Yes	Yes	Yes	No	14/16	High
3	SÃ¡nchez, 2015 [37]	Yes	Yes	Yes	Yes	Yes	Yes	Yes	No	14/16	High
4	Rudranaik, 2016 [27]	Yes	UC	Yes	Yes	No	No	Yes	No	9/16	Moderate
5	Arya, 2017 [28]	Yes	Yes	Yes	Yes	UC	No	Yes	No	11/16	Moderate
6	Smadi, 2017 [38]	Yes	Yes	Yes	Yes	No	No	Yes	No	10/16	Moderate
7	Sisli, 2019 [39]	Yes	Yes	Yes	Yes	No	No	Yes	No	10/16	Moderate
8	PÃ©rez, 2020 [40]	Yes	Yes	Yes	Yes	Yes	Yes	Yes	Yes	16/16	High
9	Sarmento, 2020 [31]	Yes	UC	Yes	Yes	No	No	Yes	No	9/16	Moderate
10	Silva, 2021 [41]	Yes	Yes	Yes	UC	UC	No	Yes	No	10/16	Moderate
11	Loureiro, 2022 [32]	Yes	UC	Yes	Yes	No	No	Yes	No	9/16	Moderate
12	Aldoss, 2023 [34]	Yes	UC	Yes	Yes	No	No	Yes	No	9/16	Moderate
13	Sarmento, 2023 [33]	Yes	UC	Yes	Yes	No	No	Yes	No	9/16	Moderate
14	Davidović, 2024 [29]	Yes	Yes	Yes	Yes	Yes	Yes	Yes	Yes	16/16	High
15	Marica, 2024 [42]	Yes	Yes	Yes	Yes	Yes	No	Yes	No	12/16	Moderate
16	Dhamija, 2025 [35]	Yes	Yes	Yes	Yes	Yes	No	Yes	No	12/16	Moderate
17	Sălceanu, 2025 [43]	Yes	Yes	Yes	Yes	No	No	Yes	Yes	12/16	Moderate
18	Barros, 2025 [44]	Yes	Yes	Yes	Yes	No	No	Yes	Yes	12/16	Moderate

Q1-Q8 definitions: Q1: Inclusion criteria clearly defined; Q2: Study subjects and setting described; Q3: Exposure measured validly and reliably; Q4: Standard criteria for condition measurement; Q5: Confounding factors identified; Q6: Strategies to address confounding factors; Q7: Outcomes measured validly and reliably; Q8: Appropriate statistical analysis.

**Table 3:** Table Summary of immune-inflammatory, proteomic, and microbiological findings in patients with PGC.

Category	Biomarker / Finding	Studies Reporting No Significant Difference (P>0.05)	Studies Reporting Significant Difference (P<0.05)
1. Inflammatory and immune-related markers	RANKL	1 [31]	-
	MMP-9	1 [31]	-
	PTHrP	1 [31]	-
	IL-17	1 [34]	1 [33]
	IL-1Î²	2 [33, 35]	-
	IL-6	-	1 [35]
	TNF-Î±	1 [33]	1 [35]
	hsCRP	-	1 [35]
2. Proteomics	Upregulated proteins (n=43)	-	1 [32]
	Downregulated proteins (n=22)	-	1 [32]
	Proteins with no significant change (n=59)	1 [32]	-
3. Microbiological analysis	Bacterial load	1 [34]	1 [44]
	Endotoxin levels	-	1 [44]

RANKL: Receptor activator of nuclear factor kappa-B ligand; MMP-9: Matrix metalloproteinase-9; PTHrP: Parathyroid hormone-related protein; IL-17: Interleukin-17; IL-1β: Interleukin-1 beta; IL-6: Interleukin-6; TNF-α: Tumor necrosis factor-alpha; hsCRP: High-sensitivity C-reactive protein.

## Data Availability

All data generated or analyzed during this study are included in this published article and its supplementary information files. The full search strategy is available upon request from the corresponding author.
